# The melding of nanomedicine in thrombosis imaging and treatment: a review

**DOI:** 10.4155/fso.16.3

**Published:** 2016-03-23

**Authors:** Varvara Karagkiozaki, Foteini Pappa, Despoina Arvaniti, Anestis Moumkas, Dimitrios Konstantinou, Stergios Logothetidis

**Affiliations:** 1 Nanomedicine Group, Lab for Thin Films – Nanosystems & Nanometrology (LTFN), Physics Department, Aristotle University of Thessaloniki, GR-54124, Greece

**Keywords:** atomic force microscopy, nanomedicine, nanoparticles, thromboembolic diseases, thrombosis

## Abstract

Thromboembolic diseases constitute a plague in our century, wherein an imbalance of hemostasis leads to thrombus formation and vessels constriction reducing blood flow. Hence, the recent rise of nanomedicine gives birth to advanced diagnostic modalities and therapeutic agents for the early diagnosis and treatment of such diseases. Multimodal nanoagents for the detection of intravascular thrombi and nanovehicles for thrombus-targeted fibrinolytic therapy are few paradigms of nanomedicine approaches to overcome current diagnostic treatment roadblocks and persistent clinical needs. This review highlights the nanomedicine strategies to improve the imaging and therapy of acute thrombi by nanoparticles and nanotheranostics, the detailed imaging of thrombogenic proteins and platelets via atomic force microscopy with the knowledge basis of thrombosis pathophysiology and nanotoxicity.

## Abstract (lay abstract)

**Lay abstract:** The present review highlights the perspectives of nanomedicine in enhancing the diagnostic and therapeutic strategies to deal with thrombosis. The basics in thrombosis are highlighted to provide the reader with better comprehension of the application of nanotools and various multimodal nanocarriers for diagnosis, targeted therapy and monitoring of the disease. The visualization and treatment of acute thrombi using multifunctional nanoparticles and nanotheranostics, along with the structural investigation of the blood-clotting proteins exploiting the atomic force microscopy capabilities are comprehensively described. At the same time, toxicity and biocompatibility issues regarding nanoparticles are discussed.

**Figure 1.**
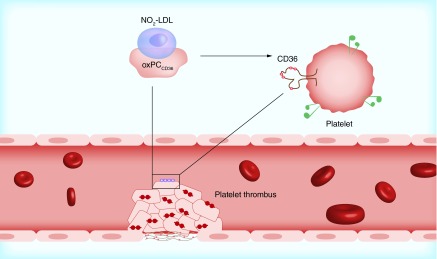
**Schematic presentation of hemostasis involving its main stages of vascular spasm after vessel injury, platelet adhesion, activation and aggregation and clot formation.** LDL: Low-density lipoprotein. Reprinted with permission from [6] © Macmillan Publishers Ltd (2007).

**Figure 2.**
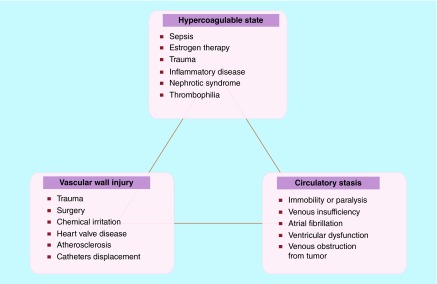
**Virchow's triad including the three main parameters that induce thrombus formation.**

**Figure 3.**
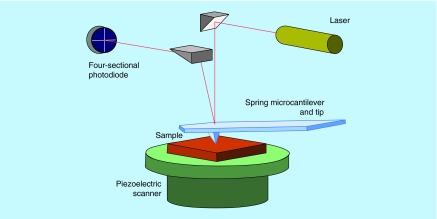
**Schematic of an atomic force microscope with optical detection of the deflection of the micro cantilever.** Reprinted with permission from [28] © Macmillan Publishers Ltd (2004).

**Figure 4.**
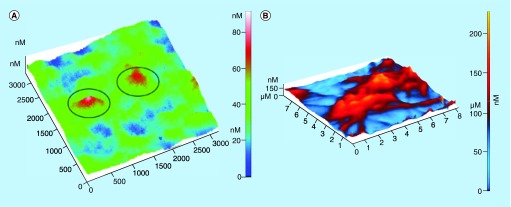
**Atomic force microscopy as functional tool for imaging of platelets’ activity.** **(A)** 3D atomic force microscopy topography image of platelets (in circles) on carbon nanocoatings after 15 min of incubation, at an early stage of activation, **(B)** after 2 h of incubation, interconnected with pseudopodia. Reproduced with permission from [29].

**Figure 5.**
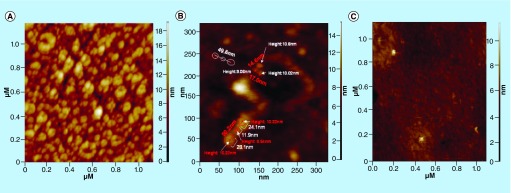
**Atomic force microscopy topography images of fibrinogen on a-C:H thin film.** **(A)** After 5 min incubation time at room temperature with encircled molecular cluster features of fibrinogen. **(B)** After incubation time of 70 min at room temperature, indicative of the length, height and conformation of Fib molecules and **(C)** Fib denaturation after adsorption at 116°C. Reproduced with permission from [31].

**Figure 6.**
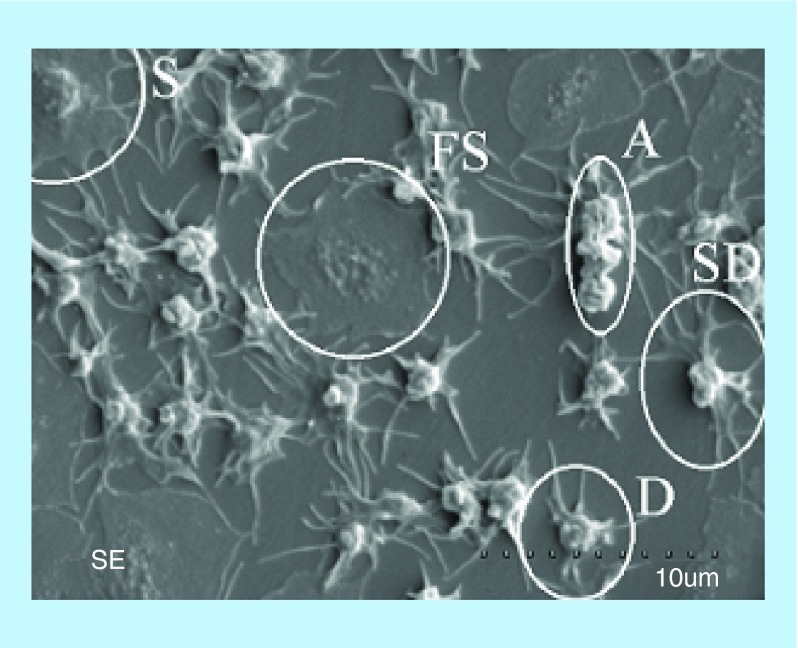
**Scanning electron microscopy image of platelets adhered to the surface of medical steel.** Different morphological forms of activated platelets are indicated. A: Platelet aggregate D: Dendritic; FS: Fully spread; S: Spread; SD: Spread dendritic. Reproduced with permission from [35].

**Figure 7.**
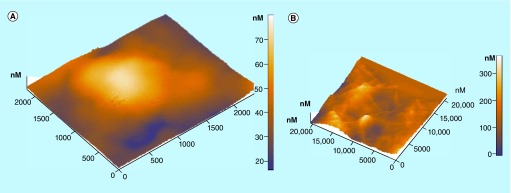
**Differentiation behavior of activated platelets on nanomaterials.** Atomic force microscopy topography images of **(A)** in-activated platelet and **(B)** highly activated platelets with pseudopodia connecting with each other onto a nanomaterial. Reproduced with permission from [36].

**Figure 8.**
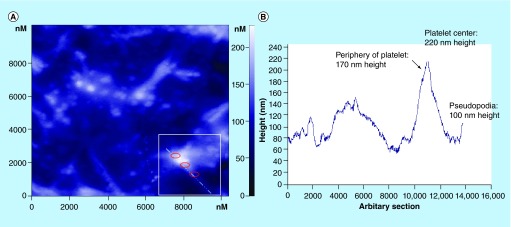
**Atomic force microscopy topography image of activated platelets onto titanium-based nanomaterial.** **(A)** An arbitrary section crossing an activated platelet with pseudopodia (within the white square) is denoted with the corresponding diagram **(B)** showing the height of the platelet's periphery, center and pseudopodia. Reproduced with permission from [29].

**Figure 9.**
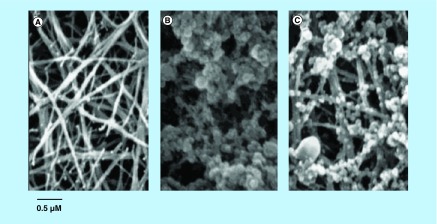
**Scanning electron microscopy images of thrombi.** **(A)** Untreated clot characterized by a dense network of fibrin strands. **(B)** Treated clot, in which fibrin strands were covered with numerous paramagnetic nanoparticles. **(C)** Partially blocked clot. Clot was pretreated with nonbiotinylated antifibrin antibody. As a result, a significant fraction of the fibrin binding sites was unavailable for binding to the targeted contrast agent. The amount of paramagnetic nanoparticles was significantly reduced as compared with the treated clot. Reproduced with permission from [42].

**Figure 10.**
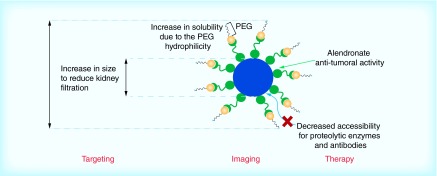
**γFe_2_O_3_ at alendronate-polyethylene glycol nanoplatform nanoparticles showing the covalent attachment of the drug to the polymer.** PEG: Polyethylene glycol. Reproduced with permission from [52].

**Figure 11.**
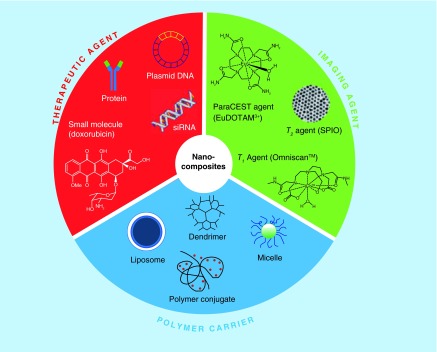
**Schematic illustration of the functional components for the development of nanomedicine showing the coating of nanoparticles surface area with specific targeted molecules against biomarkers of the disease.** Reproduced with permission from [59].

**Figure 12.**
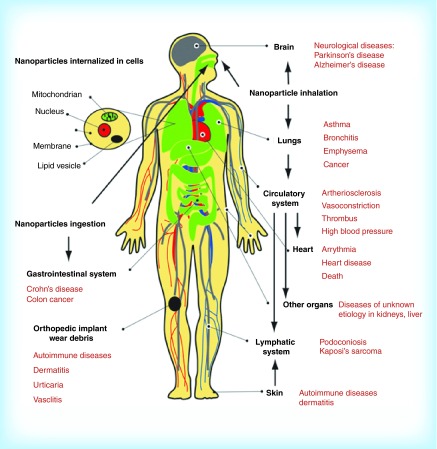
**Pathways of exposure to nanoparticles and associated diseases as suggested by epidemiological, *in vivo* and *in vitro* studies.** Reproduced with permission from [66].

Medicine is constantly evolving and new technologies are anticipated to integrate into clinical practice. Introduction of nanotechnologies in medicine will not create a separate branch of medicine but it implies the advances that can bring to diagnosis and treatment of various diseases, such as thromboembolic ones [1,2].

Thrombosis underlies a number of cardiovascular syndromes, including Acute Coronary Syndrome (ACS; the clinical spectra ranging from stable/unstable angina to myocardial infarction), stroke and pulmonary embolism that are the leading causes of morbidity and mortality in western societies [3]. Despite the detection of thrombi formation with diagnostic regimens such as Doppler ultrasound, computer tomography (CT) or MRI, there is still in clinical practice a lack of imaging modalities able to deliver information on the pathogenesis of thrombosis or on the thrombi age and their constituent components, information vital for effective treatment. Such information becomes particularly important in the case of blood-contact implants, such as vascular stents and cardiac valves where the sudden onset of thrombus formation onto their surfaces may cause arterial occlusion, heart failure and even cardiac arrest. In every day practice, angioplasty with stent implantation is the golden standard in the treatment of arterial stenosis [4]. Bare metal stents and drug-eluting stents (DES) have been used as the appropriate vehicles for site-specific drug delivery in order to circumvent restenosis. However, few clinical trials showed that the polymeric coatings of stent's surface as well as the release of antiproliferative drugs may cause hypersensitivity reactions of the arterial wall and inhibition of the stent re-endothelialization process inducing late stent thrombosis [5].

Recent rapid advances in nanotechnology and nanoscience offer a wealth of new opportunities for diagnosis and therapy of various diseases (e.g., cardiovascular disease, pulmonary and hematological diseases). The introduction of nanotechnologies in medicine involving nanoscale-structured materials like nanoparticles (NPs) holds great promise for advanced biosensors and implants, targeted drug delivery and tissue engineering [6]. Given the inherent nanoscale size of the functional components of living cells, the design and manufacturing of nanostructures similar with them in size, enables the study and control of cellular functions combined with site-specific drug delivery [7]. More precisely, in the size range of 10–100 nanometers, the NPs exhibit a large surface area to volume ratio that enables the conjugation of a wide range of therapeutic and diagnostic agents and their multiple interaction with cell membrane receptors, peptides, among others. Their small size allows them to navigate *in vivo* through blood vessels and thus deliver their cargo at the diseased site [7]. This review makes a citation of the pathogenesis of atherothrombosis, the key role proteins and cells of this procedure that are responsible for the clotting of vascular vessels and highlights how nanomedicine can advance mainly the diagnosis and treatment of thrombosis by the implementation of highly sensitive diagnostic tools and nanovectors with multifunctional activities and properties.

## Basics in thrombosis

### Hemostasis & coagulation process

**Hemostasis** is the physiological mechanism that prevents significant blood loss after a vascular injury. It comprises both the clotting process and the subsequent fibrinolysis as these mechanisms overlap each other with the additional contribution of a vasomotor response. It occurs through the rapid formation of an impermeable platelet and fibrin plug (hemostatic thrombus) at the site of injury. In order to insure a well-formed stability of the loose platelet plug, a fibrin mesh called clot, is formed and entraps the plug as shown in Figure 1. In case that the plug contains only platelets, is being renamed to white thrombus and in case of red blood cells, it is called red thrombus [8–10].

In addition, fibrin within the thrombus triggers its own dissolution by plasmin-mediated fibrinolysis, which further limits thrombus propagation. Maintenance of blood fluidity and the ability to prevent blood loss reflects therefore a delicate balance among tightly regulated platelet function, coagulation and fibrinolysis (hemostatic balance). Disturbances in the regulation of the balance may cause the formation and deposition of too little fibrin at the site of injury, resulting in impaired hemostasis – ultimately manifesting as bleeding – or enhanced fibrin formation and deposition – causing thrombosis [11].

#### The cascade model of blood coagulation

The waterfall/cascade model proposed that fibrinogen formation occurs through a series of reactions in which serine proteases, which normally circulate as inactive zymogens, are converted into active enzymes. In 1992, Mann proposed the ‘cell-based model’ where coagulation occurs in three overlapping phases: initiation, amplification and propagation. The process starts on tissue factor (TF)-exposing cells, and continues on the surfaces of activated platelets where TF, complexed with factor VIIa, is now known to be the major initiator of *in vivo* coagulation, followed by massive amplification of thrombin generation by the prothrombinase complex (factor Xa and factor Va) [10–14].

#### Initiation

Coagulation is initiated when TF also called thromboplastin or FIII, normally segregated from the flowing blood, is exposed to plasma, binding coagulation factor (F) VII/VIIa and forming a complex on cellular surfaces that triggers the coagulation cascade [10,11] whenever the integrity of the vessel wall is breached. The expression of TF can also be induced in monocytes and, to some extent, in endothelial cells in response to various stimuli, including inflammatory cytokines, endotoxin, growth factors and oxidized/modified low-density lipoproteins [10,11]. The proteolytic TF/FVIIa complex activates small amounts of FIX and FX. On TF-expressing cells, FXa then associates with FVa to form the prothrombinase complex. The prothrombinase complex cleaves prothrombin to generate small amounts of thrombin, the enzyme responsible for fibrin formation [10–12].

#### Amplification phase

The small amount of thrombin generated in the initiation phase can diffuse away and have widespread effects on multiple areas of coagulation. Referred to as the amplification phase, this prothrombotic ramping-up period results in activation of platelets with exposure of membrane phospholipids, creation of a procoagulant membrane and the release of granule contents. Thrombin cleaves FXI to FXIa and FV to FVa on the platelet surface. It also cleaves von Willebrand factor (vWF) off of FVIII to release FVIII and then activates FVIII to FVIIIa [11].

#### Propagation phase

Along with FVIIIa, FIXa binds to an appropriate membrane, usually that of the platelet, forming the tenase complex. The ‘ten’-ase complex activates factor ‘ten’, resulting in a rapid generation of FXa. This comprises FVIIIa, FX, FIXa and calcium. The tenase complex is thought to be 50-times more efficient in terms of activation of FX than the TF-FVIIa complex [11]. Activated FX initiates assembly of the prothrombinase complex, which comprises FXa, FVa and calcium. The prothrombinase complex activates prothrombin to thrombin, resulting in what is considered an explosive generation of thrombin, and subsequent fibrin clot formation. Fibrin protofibrils are formed by polymerization of soluble fibrin monomers. They are stabilized by FXIIIa (which is also activated by thrombin), to form a solid fibrin network that in turn stabilizes platelet aggregates to form a platelet/fibrin thrombus. Owing to the fact that coagulation comprises a series of enzymatic processes, an amplifying cascade results in thrombin generation; approximately one molecule of FXa generates approximately 1000 thrombin molecules of thrombin [10,11,13]. Coagulation must be dampened once a stable clot is formed at the damaged endothelium site. Healthy endothelium located downstream from the clot does not support coagulation. Thrombin cannot be generated from FXa that diffuses away from the clot – endothelial cell surface anticoagulants, AT and TFPI inhibit any FXa and thrombin that diffuse away. Thrombin can also bind to thrombomodulin, which results in loss of thrombin's procoagulant activity [10–13].

#### Fibrinolysis

After the hemostatic plug has served its main purpose by preventing the blood loss during vessel repairment, an enzymatic process called fibrinolysis occurs. Fibrinolysis is a commonly normal enzymatic cascade process of degrading the fibrin network into soluble products. The core role of this procedure, lies to the prevention of blood clots from growing and thus become more fatal by the function of key role enzyme plasmin. An abundance of fibrinolysis inhibitors keeps it quiescent in flowing blood when healthy. Fibrinolysis also requires fibrin as a co-factor for optimal function, which serves to inhibit fibrinolysis in the absence of clot formation [11].

### Overview of the pathophysiology of atherothrombosis

Thrombosis is a process where a blood clot is formed within an artery or a vein. If a thrombosis episode occurs in a coronary artery (coronary thrombosis) that will cause the artery to narrow resulting in myocardium ischemia. Coronary artery disease (CAD) is the most common form of cardiovascular disease. In CAD, atherosclerosis results in damage of the coronary artery wall and in the creation of focal lesions at sites of turbulence in arteries. These lesions may rupture, leading to thrombosis and complete occlusion of the vessel, causing myocardial infarction, stroke or acute ischemia. Atherothrombosis, a broader term includes both atherosclerosis and its thrombotic complications. The mechanism responsible for the sudden conversion of a stable stenotic atherosclerotic disease to a life-threatening condition is usually plaque disruption with superimposed thrombosis. The vulnerable high risk plaques for spontaneous rupture are characterized by the large lipid core and thin fibrous cap, the presence of inflammatory cells and the proteolytic activity in the fibrous cap [4]. Recent evidence indicates that the arterial thrombi in Atherothrombosis apart from platelets and fibrin, are composed of erythrocytes along with white blood cells, and are constitutively immunopositive for GPIIb/IIIa, glycophorin A, vWF and neutrophils [14].

Atherosclerotic plaque rupture exposes the subendothelial matrix and releases prothrombotic factors. This leads to localized platelet adhesion and activation which results in the membrane exposure of phosphatidyl serine, providing binding sites for coagulation factors. Thus, the inhibition of endogenous phosphodiesterase and cyclooxygenase may play an important role in the treatment of peripheral and cerebrovascular disease, whereas antiplatelet therapy with clopidogrel and aspirin is the cornerstone of ACS and poststent treatment.

Plaque disruption appears two main patterns: plaque rupture and plaque erosion. Plaque rupture is caused by fibrous cap disruption, and mainly allows blood to come in contact with the thrombogenic necrotized core resulting to thrombosis [15]. Thrombotic occlusion is less common with plaque erosion than plaque rupture, whereas micro embolization in distal small vessels is more common with plaque erosion than plaque rupture [16]. Accumulating evidence supports a key role for inflammation, low shear mechanical stress and abundancy of active TF in the pathogenesis of plaque rupture [17]. Although it is well recognized that mechanical stress (hemodynamic shear stress, turbulent pressure fluctuation, sudden increases in intraluminal pressure and tensile stress concentration within the wall of the lesion) triggers the disruption of fibrous cap, it remains unclear which factor is mainly responsible for this disruption [18]. The differences between the atherosclerotic and the normal artery lie to the presence of abundant active TF in atherosclerotic lesions. It has been found that even a small amount of TF in the blood is capable of supporting clot formation *in vitro* [19]. More than 150 years ago, Virchow first described the mechanism of thrombus formation. It has still remained as a fundamental theory of thrombus formation till today (Figure 2).

#### Clinical manifestations

Atherothrombosis is a predisposing condition to various diseases such as: CAD, angina, myocardial infarction and even cardiac arrest; Cerebrovascular disease: stroke, ischemic attack; Aortic aneurysms, aortic dissection; and Peripheral arterial disease [8]. Alterations in any of Virchow's triad components (blood composition, blood flow and vessel wall) can predispose to the development of deep vein thrombosis syndrome and subsequent pulmonary embolism [19].

### Cardiac implant-associated thrombosis

Thrombosis is one of the most common problems associated with prosthetic heart valves, especially with those made from metal. Although the mechanical valves are very durable, their main disadvantage is the risk of blood clots forming on valve components. Such thrombi formation can cause valve occlusion, or the thrombi can be liberated and lead to stroke or myocardial infarction [20]. Hence, thrombosis can also happen in rare cases of patients who received bioprosthetic heart valves, according to a case study released in 2009 in the online publication ‘*Circulation*’ by the American Heart Association [21]. The risk factors associated with the formation of thrombus include a lack of anticoagulant medications for the postoperative treatment of the patient and the presence of possible coagulation disorders that may predispose a patient to thrombus formation on the valve. Open-heart surgery is usually the management of choice for patients that present with this kind of complication. It is necessary for all patients with mechanical valves to receive a lifelong anticoagulant therapy and their treatment regimen should be closely monitored. Inadequate anticoagulation leads to blood clotting, while excess therapy can cause dangerous internal bleeding or blood loss from minor injury [22]. For several years, restenosis was the Achilles’ heel of coronary artery stenting that limited its long-term efficacy. Introduction of DES reduced restenosis whereas recent studies have shown a higher incidence of stent thrombosis and particularly of late stent thrombosis [23]. According to Academic Research Consortium (ARC), a new standard definition of stent thrombosis has been proposed in order to compare the true rates of stent thrombosis across different trials and registries [24]. The ARC is composed of clinical investigators, industry representatives and regulators including the US FDA.

This definition categorizes stent thrombosis according to the level of documentation and timing:

Definite or confirmed event (symptoms suggestive of an acute coronary syndrome and angiographic or pathologic confirmation of stent thrombosis);
Probable event (unexplained death within 30 days or target vessel myocardial infarction without angiographic confirmation of stent thrombosis); and
Possible event (any unexplained death after 30 days post stent implantation).

Based on the elapsed time since stent implantation, stent thrombosis can be classified as:

Early (0–30 days post-stent implantation);
Late (>30 days);
Very late (>12 months).

Often, early stent thrombosis is further subdivided into acute (<24 h) and subacute (1–30 days) events. The mechanisms of stent thrombosis have not been fully explored yet, but both bare-metal stents and DES were found to induce platelet adhesion and thrombus formation and therefore, dual antiplatelet therapy is mandatory for some time in stent patients [25]. After stent implantation, endothelial cells cover its surface to prevent clot formation. The cytotoxic drugs used in DES to reduce SMC proliferation and the polymers that serve as drug reservoirs are considered to inhibit endothelialization process, and to cause chronic inflammation in the coronary artery characterized by infiltration of eosinophilic cells in the vessel wall and this might also contribute to a prothrombotic environment [26].

## Nanomedicine perspectives

### Atomic force microscopy as an imaging tool for thrombosis

Due to the small size of thrombogenic proteins that range from nano to micrometer, the need of observation by an imaging tool with high-resolution capacities for real-time studies of biological and nonbiological interactions seems imperative. Nowadays, an advanced technique is scanning electron microscopy (SEM) which has been widely used for imaging microscopic features on sample surfaces. SEM images provide great resolution at the range of approximately 5 nm. SEM function is based on a beam of electrons which travel to the sample in order to provide an image. To this point, nanomedicine can provide analytical tools with higher sensitivity for understanding the ongoing processes at the cellular level, leading to the elucidation of thrombosis mechanisms. Atomic force microscopy (AFM) is a representative example of such a tool and exhibits resolution lower than 1 nm. The operation of this technique involves scanning of the surface of the sample with a sharp tip in a contact area of few square nanometers of a micro-fabricated cantilever. The forces between the tip and the sample are sensed by the cantilever and the deflection is measured by a laser. Figure 3 presents the main principles of AFM operation [27]. AFM, which enables the high resolution imaging of cells and proteins at the nanoscale, can facilitate the comprehensive analysis of platelets behavior and their degree of activation in parallel with the analysis of thrombogenic proteins and the factors that affect their conformation and function (e.g., temperature), prerequisite for thrombus formation. The in-depth analysis of platelets behavior, in a native, unlabeled state, in three dimensions (providing cross-sections for more details of cell surface and features) resembling the human conditions can give us data about the cell attitude toward biomaterials for their thrombogenicity assessment. Karagkiozaki *et al*. used AFM to analyze morphological alterations of platelets during activation onto nanomaterials for cardiovascular implants [4]. Figure 4A & B depict 3D AFM images of platelets at different stages of activation. More precisely, in Figure 4A, the platelet is activated after 15 min adhesion on carbon-based nanomaterial and it loses its discoid shape becoming spherical. Figure 4B shows highly activated platelets with filopods-pseudopodia connected with each other forming a network, compromising a prestage of a thrombus.

AFM provides a number of advantages over conventional microscopy techniques. It is a unique technique, where the probing of sample is being measured in three dimensions. AFM requires neither a vacuum environment nor any special sample preparation, so it can be used in a various range of environments. Thus, it enables imaging of proteins, peptides, chromosomes, cells and other biological samples of 3D structure and morphology, their interactions, and dynamic configurations during motion [4].

Fibrinogen (Fib) is a key protein for thrombus formation as it serves as intercellular glue between platelets by binding to plasma membrane of platelets mediating platelet aggregation [28]. Fib is a linear blood plasma protein with three globular domains. Heating or pH change may cause protein denaturation due to the easy breakage of hydrogen bonds that maintain protein's shape. AFM technique can provide information about the effect of temperature on protein conformation. More precisely, in Figure 5A, topography images of Fib on amorphous hydrogenated carbon (a-C: H) thin film are presented after 5 min incubation time at room temperature (RT). The circle indicates the molecular cluster features of Fib. It was revealed that the three-lobe structures that appear are indeed, small clusters of few molecules of Fib, with either extended or V-shape conformations that still retain the shape of a single molecule [29]. In Figure 5B, the AFM topography image of Fib adsorbed on a-C: H thin film, after incubation time of 70 min is presented [30]. Previous work from our laboratory showed that by using the heating stage of AFM, Fib molecules unravel and the 3D structure of the protein is altered [31,32]. For example, topography image of Fib on amorphous carbon thin film at 116°C compared with Fib adsorption at RT is depicted in Figure 5C. It can be easily noticed that Fib molecule at this temperature changes from a linear to a folded conformation.

Moreover, while recognition of individual proteins such as thrombogenic ones, is still a challenge, the technique to functionalize an AFM probe with specific molecules such as antibodies establishes a promising way to identify such proteins and this AFM function enables the thrombosis elucidation. It has been shown that individual receptors can be identified by an AFM tip functionalized with antibodies through a force mapping technique or directly from a phase image in tapping mode [33].

AFM enables the observation of platelets in a native, unlabeled state for several hours without damage. By their analysis one can conclude whether the platelets are at an early or later stage of activation and have tendency for aggregation and clot formation. The early stage of platelet activation is characterized by change of their shape into more spherical and while activation continues, a cascade of structural changes takes place; composition of pseudonucleus due to the gathering of their alpha-granules and dense ones into the center of the cell (transforming into egg-like type cell); formation of fully spread pseudopodia. This procedure normally ends in thrombosis and even embolism. Based on the shape of cells, Goodman categorized platelets into five morphological types arranged according to the increasing stage of their activation [34]. These are, in the sequence of activation degree, discoid (round), dendritic (early pseudopodia), spread dendritic (intermediate pseudopodia), spread and fully spread platelets. Figure 6 depicts an SEM image of platelets adhered to the surface of polished medical steel with different morphological forms of cells in line with the degree of their activation.

AFM can also provide details with nanoscale precision of these morphological alterations of platelets during activation as shown in Figure 7. Especially, 3D, AFM topography images of platelets onto nanomaterials, in sequence with the increasing stage of activation, are presented: the platelet at early stage of activation has become spherical with height value about 70 nm (Figure 7A), and, highly activated platelets having egg-like type morphologies with pseudopodia that form aggregation (Figure 7B) [3,35].

Details in AFM imaging are more pronounced if crossing sections of the topography images are performed. For instance, by performing an arbitrary section crossing an activated platelet with pseudopodia, the height of its components can be measured (Figure 8). It was found that the height of platelet periphery was 170 nm, of its center 220 nm and of its pseudopodia 100 nm (diagram of Figure 8) [37].

Overall, in this section it was showed that nanomedicine can aid in the elucidation of thrombosis mechanisms by the application of nanoscale imaging techniques for thrombosis-associated proteins visualization, for better comprehension of their conformations under different stimuli and by detailed observation of platelets during activation and aggregation.

### Nanoparticles as catalytic agents for thrombosis imaging & treatment

#### Multimodal nanovectors for detailed imaging of thrombosis

Acutely formed thrombi are the causal agents in life-threatening clinical syndromes such as myocardial infarction, stroke and pulmonary embolism. Until now, imaging modalities in clinical practice that allowed thrombi visualization include ultrasound, x-ray, CT or MRI. However, there is lack of information about thrombus composition and age that enables an effective treatment with thrombolysis and anticoagulants. Therefore, a number of molecular imaging strategies have been developed in order to image thrombus formation, including fluorescently labeled platelets [38] and fluorescently or radio-labeled ligands targeted to other components of thrombus such as fibrin [39] and coagulation factors [40]. Nanomedicine entails numerous engineered constructs, assemblies, architectures and particulate systems used for diagnostics and targeted drug delivery, whose unifying feature is their nanoscale size range. These include polymeric micelles, dendrimers, polymeric and ceramic NPs, protein cage architectures, viral-derived capsid NPs, polyplexes and liposomes that release diagnostic and therapeutic payloads *in situ*.

#### Fibrin-targeted contrast nano-agents

The sensitivity of a novel fibrin-targeted contrast agent for fibrin detection was defined *in vitro* on human thrombus. The contrast agent was a lipid-encapsulated perfluorocarbon NP with numerous gadolinium-diethylene-triamine-pentaacetic acid (Gd-DTPA) complexes incorporated into the outer surface. After binding to fibrin clots, SEM image of treated clots revealed dense accumulation of NPs on the clot surfaces (Figure 9). Fibrin clots with sizes ranging from 0.5 to 7.0 mm were imaged on a 4.7 Tesla magnet with or without treatment with the targeted contrast agent. It was found that regardless of size, the untreated clots were not detectable by MRI, while targeted contrast agent dramatically improved the detectability of all clots. The researchers suggest the potential for sensitive and specific detection of micro thrombi that were formed on the intimal surfaces of unstable atherosclerotic plaque leading to definitive early diagnosis and initiation of preventative therapies for thromboembolism [41].

#### EGF-targeted NPs

Mei *et al*. assessed the effect of EGF1 in directing NPs to thrombi [28]. The synthesized EGF1 peptide was conjugated to NPs forming EGF1–NP. A lipophilic fluorescent dye, coumarin-6, was incorporated into EGF1–NP in order to detect its loading and release capacity. The binding ability of EGF1–NP with TF-expressing cells was shown to be significantly higher than that of the nonconjugated ones. Following an intravenous administration, fluorescence was distributed along the vessel wall of thrombotic regions in the model rats injected with coumarin-6-loaded EGF1–NPs. The *in vitro* and *in vivo* results suggest that EGF1–NPs constitute a promising drug delivery system for targeting cerebral thrombi [42].

## Nanocarriers for thrombosis treatment

Current nanomedicine strategies for thrombosis target to replace or combine with previous pharmacological approaches. Recent advances in nanodevices have been largely achieved by either combining older treatment methods with new NP technology, or by implementing entirely novel techniques that have shown significant promise in the field. Nowadays, this ‘branch’ of medicine has been applied, at least at experimental stages with clinical applications for thrombosis [43].

### Liposomes as drug delivery systems

First-generation NP systems, such as liposomes, constitute one of the most suitable carriers for drugs and nucleic acid therapeutics. They exhibit different characteristics which affect their effectiveness such as size, chemical structure and shape. These engineered, multifunctional drug delivery nanosystems can simultaneously treat and monitor diseases through the inclusion of many different diagnostic and therapeutic agents within a single formulation. Palekar *et al*. functionalized the surface of liposomes with multiple copies of the direct thrombin inhibitor d-phenylalanyl-l-prolyl-l-arginyl-chloromethyl ketone (PPACK), which exhibits high affinity for thrombin as a free agent but manifests too rapid clearance *in vivo* to be effective alone [44]. Viruses also constitute promising nanocarriers [45]. Table 1 depicts a few of the major nanocarriers and their therapeutic activity in thrombosis.

### Synthetic polymer nano-carriers

A variety of polymer combinations for drug delivery systems (DDS) have been employed in clinical trials. The major advantage of these systems is the diversity of shapes that can be achieved whereas the polymeric architecture plays a critical role in their effectiveness [50]. Small changes in architecture might have an effect on solubility, drug loading capability and biodegradability. The drug can be physically incorporated to the matrix or shell of the polymer, or can be covalently attached to the polymer itself, as it is shown in the schematic representation of γFe_2_O_3_ at alendronate-polyethylene glycol (PEG) NPs in Figure 10 [51]. A distinct advantage of polymer combinations is that due to their nanoscale size the incorporated drugs can be delivered much more efficiently. Recent activity has designed and conjugated thrombin-sensitive peptide substrates to the surface of NPs in order to detect thrombi in living organisms. Similar to liposome delivery, another proven strategy to improve NP delivery is PEGylation that can reduce the opsonization effect and NPs uptake by macrophages. The PEG coating not only attenuates the activation of host immune response, but also improves pharmacokinetics [52].

### Thrombin-inhibitor NPs

In clinical practice, there is an urgent need for new potent and highly specific antithrombotic agents with minimal toxicity for treatment of thrombosis in ACS, stroke, venous thrombosis and stent placement. As a regulator of the penultimate steps in the coagulation cascade, thrombin, a serine protease, represents a principal target of direct and specific anticoagulants for localized control of acute thrombosis. Myerson *et al*. have recently shown that thrombin-inhibiting perfluorocarbon NPs provide a novel strategy for treatment and imaging of acute thrombosis [46]. A potent thrombin inhibitor complexed with a colloidal NP was engineered as a first-in-class anticoagulant with prolonged and highly localized therapeutic impact conferred by its multivalent thrombin-absorbing particle surface. PPACK was covalently secured to the surface of perfluorocarbon-core NP structures. The PPACK NP inhibition of thrombin was assessed both *in vitro* via thrombin activity against a chromogenic substrate and *in vivo* through intravenously administration prior to acute photochemical injury of the common carotid artery. Perfluorocarbon particle retention in extracted carotid arteries from injured mice was assessed via magnetic resonance spectroscopy and MRI. This study resulted that PPACK NPs exceeded PPACK's intrinsic activity against thrombin and that they outperformed both heparin and uncomplexed PPACK in inhibiting thrombosis. Magnetic resonance spectroscopy confirmed that PPACK NPs specifically bound to sites of acute thrombotic injury. The researchers conclude that PPACK NPs present thrombin-inhibiting surfaces at sites of acutely forming thrombi and they suggest them as a new platform for localized control of acute thrombosis [65].

### Nanoparticle-protein conjugates

Nanoparticle-protein conjugates represent another example where a complex is synthesized through PEGylation [53]. In particular, new trends in polymer conjugate technology including site-specific modification following protein mutagenesis, selective glutamine PEGylation in proteins via utilization of the enzyme transglutaminase and using degradable PEG-protein linkages have been investigated, in order to maximize protein bioactivity. In acute vascular events, the endothelium derived TF is the trigger of the coagulation cascade. In a recent study, EGFP–EGF1 protein-conjugated PEG-PLGA NP was employed as a TF targeting vehicle. NF-κB decoy oligonucleotides (ODNs) were incorporated into it and the resulting EGF1–EGFP–NP-ODNs were evaluated as a vector for therapy of cortex infarction. It was found that 6 h after intravenous administration *in vivo*, most EGF1–EGFP–NPs were accumulated in the embolism vessels, distributed in damaged endothelial cells and lowered the TF expression [54]. Furthermore, animal testing at experimental ApoE^-/-^ mice, showed that elastin antibody tethered NPs (EL-NPs) gave a strong fluorescent signal in the aortic arch indicating their presence 24 h after the injection. NPs were completely absent at the healthy mice aorta [55]. Thus, such biofunctionalized NPs can be used for imaging of vascular diseases due to the fact that the elastic lamina is also connected to atherosclerosis and NP binding is proportional to that damage.

## Nanotheranostics

NPs that hold both therapeutic agents and diagnostic ligands are popularly referred to as ‘Theranostic nanoparticles’ aimed for improved diagnosis, targeted therapy and monitoring of various diseases such as thrombosis and atherosclerosis [56]. In particular, by coating the NPs surface area with specific targeted molecules against biomarkers of the disease, the specific delivery and accumulation within pathological tissue and elution of the therapeutic agent can be achieved by stimuli-responsive release [57]. In Figure 11, the major functional components for the development of nanomedicine, thus enhanced the combining area for nanotheranostics, are presented [58]. Nanotheranostics studies can roughly be classified into three distinct categories. First, the use of nanoparticulate contrast agent-aided imaging to evaluate the efficacy of therapy; second, the use of imaging to evaluate nanotherapy; third, the use of theranostic NPs for the purpose of diagnosing and treating the disease [59]. In the aforementioning study by Myerson *et al*. [46], the nanotheranostic PPACK NPs (with a final size of 160.5 nm) tested in a photochemical injury thrombosis mouse model, were found to have higher antithrombotic efficacy compared with free PPACK and heparin, as PPACK NP-treated mice developed thrombosis at later time points. Peters *et al*. developed a micelle-based theranostic NPs (17 nm size) that can be loaded with anticoagulation drugs and peptides that specifically bind to clotted plasma proteins [60]. NPs showed specific *in vivo* targeting to atherosclerotic plaques whereas when loaded with hirulog, an antithrombotic peptide, they showed better antithrombotic activity than free hirulog at the same molar concentrations in ApoE KO mice [60].

In the case of atherothrombosis and stent complications, the main target for the design of the multi-functional NPs is the ability to achieve the optimized blood-circulation time, to eliminate the clot and to reach the intra-plaque components in order to reduce plaque inflammation [61]. The injured endothelium, platelet hyperactivity, clotting, macrophage-mediated processes, matrix modelling events, coagulation factors and deregulated metabolic activities constitute the disease-specific targets [4].

Nanotheranostic approaches may have significant benefits in the simultaneous diagnosis and treatment of the acute thrombi following the plaque rupture, by monitoring the NPs biodistribution in the body over time, their localization into thrombus area, penetration through the endothelium into the ruptured plaque and their interactions with the target cells in line with the *in vivo* pharmacokinetics [4].

However, due to their complexity, it is unlikely that theranostic NPs will be consistently applied clinically. There are cases whereby the diagnosis is negative and the activity of applied nanotheranostics is eliminated and discarded through the body. Thus, in order to maximize the effectiveness of nanomedicines, the separation of diagnosis from treatment in a few cases is preferable. This is in contrast with the target of nanotheranostics that should apply simultaneously diagnosis and treatment and this difference should be addressed by researchers. Sorting out this issue and taking into account the advantages of nanotheranostics, this field could provide, in the future, a more personalized therapy rendering diseases prognostic, curable or at least treatable at their earlier stages.

## Nanotoxicity: the other side of the coin

Except of their unique advantages, NPs also present limitations. Great research and regulatory activity take place in the area of NP health, safety and toxicity [62]. The evaluation of safety risks of materials involved in nanotechnology is attributed to the field of nanotoxicology. Toxicity is an important factor that must be taken into account when the clinical translation potential of NPs is examined [63]. Nanoparticle's toxicity can be affected by many factors, such as material chemical composition, size, shape, aggregation, solubility, surface charge and structure, surface functional groups and coating material and thickness [64]. Toxicity is also influenced by the chemical composition and dose of the agent and by additional factors such as the route of administration [65]. In Figure 12 multiple pathways of NP exposure and associated diseases which were found from *in vivo* and vitro studies are presented. Because of their small size and large surface area in comparison with their volume, the danger is magnified through a greater chemical reactivity and biological activity [65]. It is known that the complement system can be activated by allergens, causing allergic reactions or even anaphylaxis and can impose human life into threat. NPs and few of their components may induce intense complement activation leading to undesired effects. Studies have demonstrated that complement activation is heavily dependent on the surface characteristics of the NPs [66–68]. Additionally, NPs into circulation may interact with elements of the immune system (such as macrophages) and cause several undesirable side effects including either immunostimulation or immunosuppression. Biocompatibility issues are also very important since degradation of by-products may lead to potential toxicity. Moreover, the activation of the immune system by DDS is of great importance and all their components must be evaluated for their biocompatibility. Since many reported applications of NPs for biological use are fairly recent, their *in vivo* distribution and toxicity profiles are still not fully understood. An array of NPs with different sizes, shapes, chemical composition and surface charge must be tested so that a complete profile of NP hazard could be provided [69]. This requires time and research. Conflictions and variations between scientific groups regarding *in vivo* behavior of various NPs need to be resolved so that their clinical translation can be established.

## Conclusion

Over the last few years there have been tremendous advances in the field of nanomedicine, mainly driven by the promising outcomes resulting from pilot studies. The usage of nanostructured materials, nanovectors and nanoscale techniques in biomedical applications offers tremendous opportunities and advances on the therapeutic and diagnostic tools in the fight against various diseases, such as thromboembolic ones. This review gives an overview of the implications of nanomedicine to advance visualization and therapy of acute thrombi by nanocarriers with diagnostic and therapeutic payloads and to elucidate interactions between platelets and thrombogenic proteins that are essential for clotting formation via AFM. The basic principles of thrombosis and cardiovascular implant-associated thrombosis are highlighted to provide evidence of the challenges that nanomedicine may evolve into a valuable tool toward new therapeutic and diagnostic regimens for thrombosis.

## Future perspective

Although nanomedicine is a relatively young field, promising outcomes resulting from pilot trials and a great number of studies have shown its growing potential. The penetration of numerous nanovectors and multimodal nanocarriers such as NPs, liposomes and several nanoscale techniques in biomedical applications offer huge opportunities and improvements for the prevention, accurate diagnosis and treatment at an early stage of thrombosis. Nanomedicine approaches enhance the traditional medicine, offer many tools and have a great potential. However, many aspects must be taken into consideration such as material requirements for NPs, effective biofunctionalization, optimal drug loading and release of the NPs, their biodistribution and ability to cross the biological barriers, their site-specific efficacy safety in line with safety. Appropriate animal models to resemble the human circulation are necessary for the valid testing of the NPs effectiveness for thrombus lysis. Maybe some issues seem to be extraordinary now, but we believe that in the coming years many of these improvements will take place and nanomedicine will become an integral part of medicine. When all those issues will be addressed for future exploration and clinical translation, such nanovectors will be promoted by human trials to validate their safety and effectiveness making them a clinical reality.

**Table 1.** **Nanoagents and their therapeutic activity in thrombosis disease.**

**Nanoagents**	**Activity**	**Ref.**
Liposomes	Anticlotting inhibitors (PPACK) against acute thrombosis	[44]
Perfluorocarbon nanoparticles	Inhibitors (PPACK) of clot-bound thrombin	[46]
Polymeric nanocarriers	Enoxaparin for antithrombotic activity	[42]
Dendrimers	Enoxaparin for attenuation of thrombi formation	[47]
Magnetic nanoparticles	Conjugation agents along with urokinase for thrombolytic activity	[48]
Iron oxide nanoparticles	Conjugation agents along with tissue plasminogen activator for thrombolysis	[49]

PPACK: d-phenylalanyl-l-prolyl-l-arginyl-chloromethyl ketone.

**Executive summary**

Tremendous advances in the field of nanomedicine took place over the last few years, mainly driven by the promising outcomes resulting from pilot studies.
Cardiovascular and thromboempolic diseases are considered to be the pandemic of 21st century, thus leading to death of one in four people daily worldwide. Thromboembolism is a significant cause of morbidity and mortality, especially in adults and the treatment may involve anticoagulants, aspirin or vasodilators. Until today, clinical medicine cannot apply effective solutions in order to successful diagnose and treat them.
Via nanomedicine's approach and the use of diverse nanoparticles, current medicine has the tremendous ability to use advanced diagnostic modalities and therapeutic agents for the early diagnosis and treatment of such diseases, which are not successfully addressed by conventional medicine.
Preclinical research conducted with lipids, polymer or magnetic nanoparticles loaded with thrombolytic drugs, targeted-liposomes showed an enhancement of thrombolysis and a reduction of undesirable side effects.
In clinical practice, the most acute treatment is the immediate dissolving of the thrombus by administration of thrombolytic drugs. As these drugs are rapidly inactivated in the blood, high amounts of them are thus injected to patients with the risk to develop intracranial hemorrhage.
Nanocarriers and drug delivery nanoparticles have been tested in preclinical models to deliver thrombolytic drugs in a site-specific targeted way. These systems have the advantage to protect the drug from the degradation and to release it at a controllable manner.
Nanomedicine can offer suitable tools in order to prevent acute thrombosis and examine the underlying mechanisms of thromboembolic diseases.
The use of atomic force microscopy as an imaging tool in order to visualize thrombogenic key factors, like platelets, and fibrinogen at the nanoscale, enables the in-depth investigation of thrombosis mechanisms.
